# Congenital herpes simplex with ophthalmic and multisystem features: a case report

**DOI:** 10.1186/s12887-023-04423-1

**Published:** 2023-12-04

**Authors:** Samuel Montenegro Pereira, Rian Vilar Lima, Maria Carolina Rocha Muniz, Marcus Breno Farias Araújo, Luiz de Moraes Ferreira Júnior, Juliana Tiburtino de Queiroz Sales Martins, Cláudia Faustino Coelho Luz, David Antônio Camelo Cid, Daniel da Rocha Lucena

**Affiliations:** 1 Waldemar de Alcantara General Hospital, Fortaleza, Brazil; 2 University of Fortaleza, Fortaleza, Brazil; 3 Ceara School of Ophthalmology, Fortaleza, Brazil

**Keywords:** Ocular herpes simplex, Neonatal HSV Infection, Pregnancy Complications, Case Report

## Abstract

**Background:**

Neonatal herpes simplex virus (HSV) infection is rare and has significant morbimortality rates. Approximately 85% of newborns are infected intrapartum, and risk factors for mother-to-child transmission include vaginal delivery, primary maternal infection, and prolonged rupture of membranes. Neonatal HSV can manifest with isolated mucocutaneous lesions, neurological involvement, or disseminated disease. In general, herpetic infection can cause blepharoconjunctivitis or keratitis. We report a rare case of congenital herpes with ophthalmologic manifestations and multisystemic involvement.

**Case presentation:**

A preterm infant, born at 32 weeks and 2 days, with presumed neonatal infection developed intestinal and respiratory complications, as well as hyperemic lesions on the left nostril and oral mucosa. An ophthalmological assessment was requested and brought up the suspicion of HSV infection, indicating empirical treatment with endovenous acyclovir. Later, a new ocular examination was suggestive of panuveitis. Afterward, serum IgM antibodies to HSV-1 and HSV-2 were positive. Proper antiviral therapy led to an improvement in the condition.

**Discussion:**

Neonatal herpes is associated with a high risk of persistent skin lesions, long-term neurological disability and other lasting sequelae. It is essential to consider HSV infection in cases of neonatal conjunctivitis, especially in patients with an epithelial defect and no improvement after initial treatment with topical or systemic antibiotics.

**Conclusions:**

In the management of neonatal HSV, early diagnosis is essential for the timely initiation of antiviral therapy. Our report highlights that ocular assessment can be crucial in the correct diagnostic investigation of this condition.

## Background

Neonatal herpes simplex virus (HSV) infection is rare and has variable epidemiology worldwide due to different birth rates and diverse viral seroprevalence [1]. Despite the important advances made in perinatal care over the years, the disease is still associated with significant morbidity and mortality [2]. The estimated annual incidence is variable, ranging between 1.6 and 8.4 per 100,000 live births [3]. Both serotypes, HSV-1 and HSV-2, are related to neonatal infections, although the risk of mother-to-child transmission is considerably higher with HSV-1 [4, 5].

Although in most cases the method of transmission is not well established, it is estimated that approximately 85% of babies are infected intrapartum, 10% in the postnatal stage and 5% in utero [5]. Most women who transmit HSV to their children have no documented history of genital herpes, either due to the absence of lesions or the subtlety of the symptoms, which leads to misdiagnosis [6]. The main risk factors associated with neonatal exposure include vaginal delivery, primary maternal infection and prolonged rupture of membranes [7].

Neonatal HSV can manifest with isolated mucocutaneous lesions, neurological involvement, or disseminated disease [8]. In most cases, these clinical syndromes have overlapping features, that is, they can develop simultaneously. Regarding ocular manifestations, overall, herpetic infection can cause blepharoconjunctivitis or keratitis, with chorioretinitis or optic atrophy being rare conditions [9]. Given the high mortality rates and potential for long-term repercussions, early diagnosis and timely antiviral treatment are essential for better clinical outcomes.

We report a rare case of congenital herpes with ophthalmologic manifestations and multisystemic involvement, highlighting the major role that ophthalmologic evaluation had in the management of the patient.

## Case presentation

The preterm male infant was born at 32 weeks and 2 days by a vaginal delivery 18 h after rupture of the membranes, with a birth weight of 2.092 g and an Apgar score of 6/8 at 1 and 5 min, respectively. The mother denied former or current use of drugs or medications but reported inadequate prenatal care, with only one medical visit made throughout pregnancy and no serology exams performed. At hospital admission, she had only a reactive VDRL result of 1:1 and revealed treatment for syphilis in prior pregnancy, raising the possibility of a serological scar. On examination, she presented only nonspecific lesions in the genital area. The clinical hypothesis of neonatal infection was brought up, antibiotic therapy was started, and infectious screening, lumbar puncture (LP), and cranial computerized tomography (CT) scan were indicated for the newborn.

**Fig. 1:**
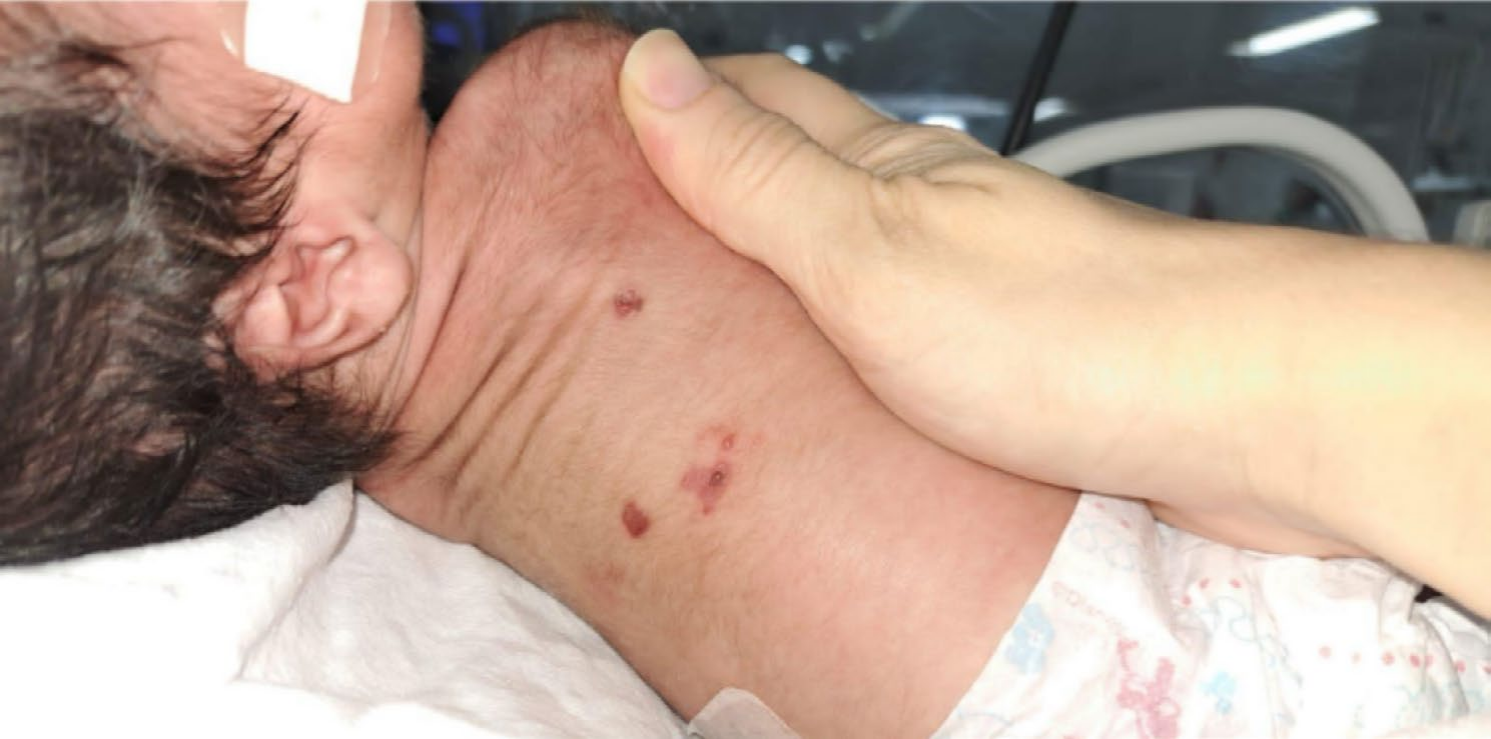
Papulopustular lesions on the back

Serial blood cultures were performed throughout the hospital stay, including on admission, but were persistently negative. Cerebrospinal fluid (CSF) analysis showed the following: proteins = 110 mg/dL; 80 cells per mm3; red blood cells (RBC) = 12,000; VDRL negative. No changes in medical management were made on account of these findings. Some days later, the baby was transferred to the neonatal intensive care unit (ICU) due to abdominal distension, bloody gastric residuals and respiratory distress. The next day, papulopustular lesions emerged on the back and chest (Fig. 1). Afterwards, the infant suffered further episodes of apnea, cyanosis and bradycardia. Thus, the antibiotic regimen was optimized for a wider spectrum, and mechanical ventilation was indicated. Later, hyperemic lesions were identified in the left nostril and on the oral mucosa.

**Fig. 2:**
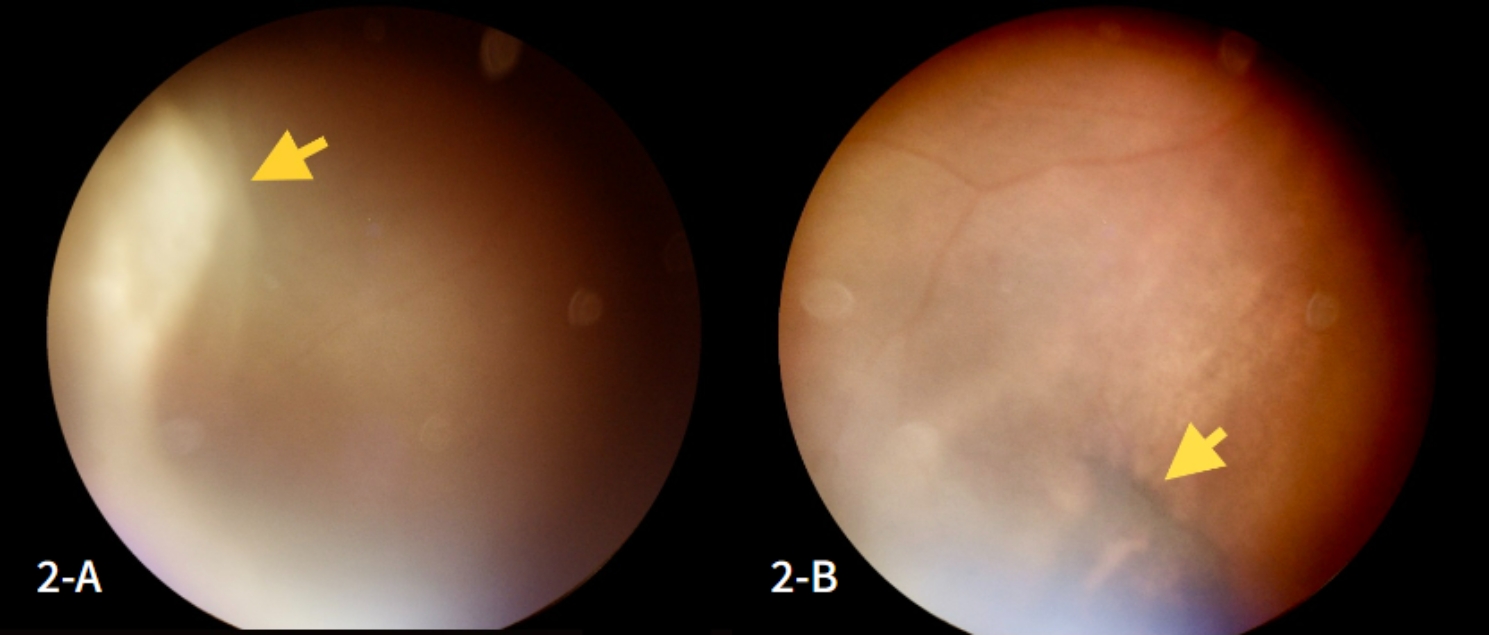
**A**: Appearance of active retinal necrosis (yellow arrow) in the right eye (RE); **2-B**: Retinography with sequelae of retinal necrosis in the RE in the same visit

**Fig. 3:**
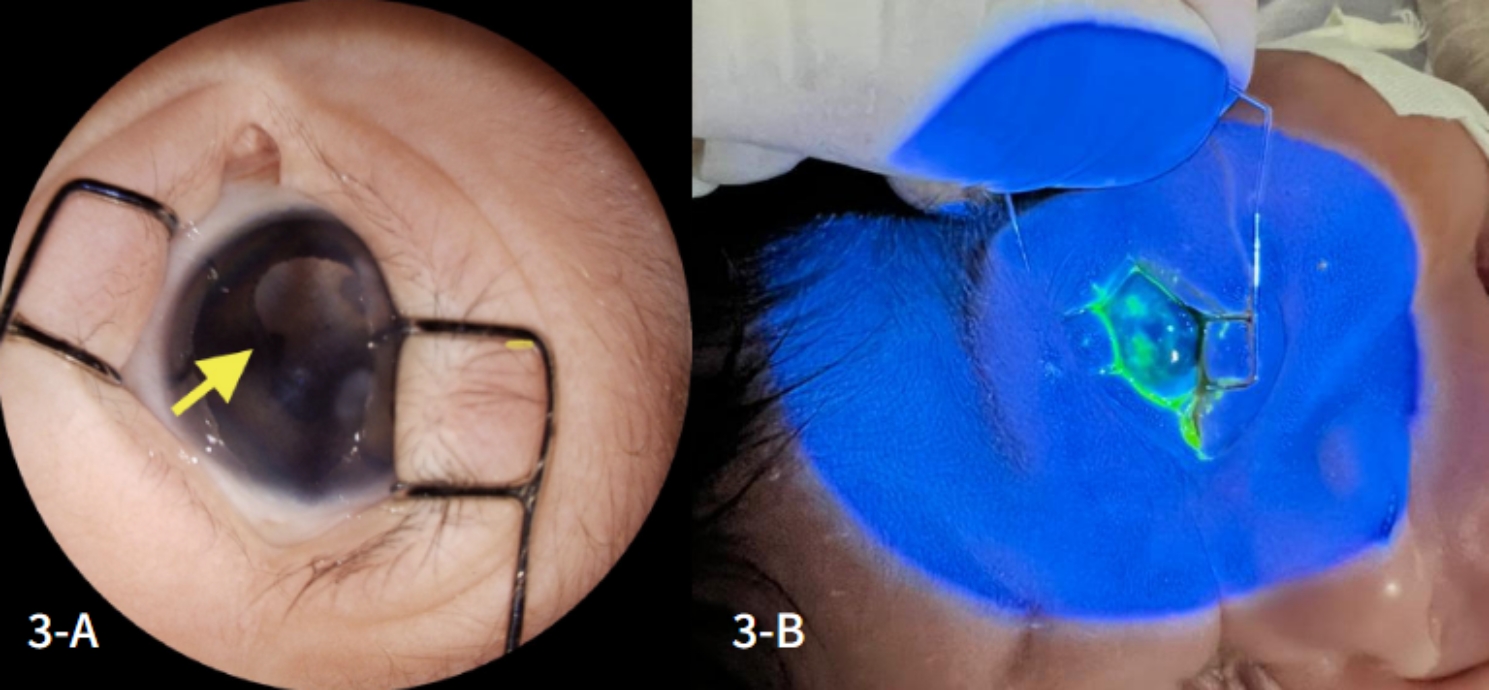
**A and 3-B**: Extensive corneal opacity and irregular, superficial, fluorescein-stained posterior synechiae, indicating epithelial keratitis with a dense infiltrate, mainly affecting the upper paracentral and peripheral regions of the left eye

An ophthalmologic assessment was requested, revealing in the right eye (RE) signs suggestive of retinal necrosis in the superior temporal region (Fig. 2-A) and pigmented areas in the inferior portion of the retina (Fig. 2-B), which was impossible to perform in the left eye (LE) due to corneal edema (1+/4+) and vitreous opacification. External eye examination detected mild hyperemia, palpebral edema, and an irregularly shaped fluorescein-stained corneal lesion in the LE, suggestive of panuveitis (Fig. 3-A and 3-B).

The suspicion of congenital neonatal infection was raised, with a strong suspicion of HSV. Empirical treatment with intravenous acyclovir was started, and additional serologies were collected to investigate toxoplasmosis, rubella, human immunodeficiency virus (HIV), hepatitis B, syphilis and cytomegalovirus. Due to the ophthalmic findings in the RE (Fig. 3-B), it was decided to start topical treatment with moxifloxacin 5 mg/ml, initially with 1 drop every 3 h for 5 days, then 1 drop every 6 h for another 10 days and artificial tears (sodium hyaluronate 0.15%) every 2 h for the same period.

**Fig. 4:**
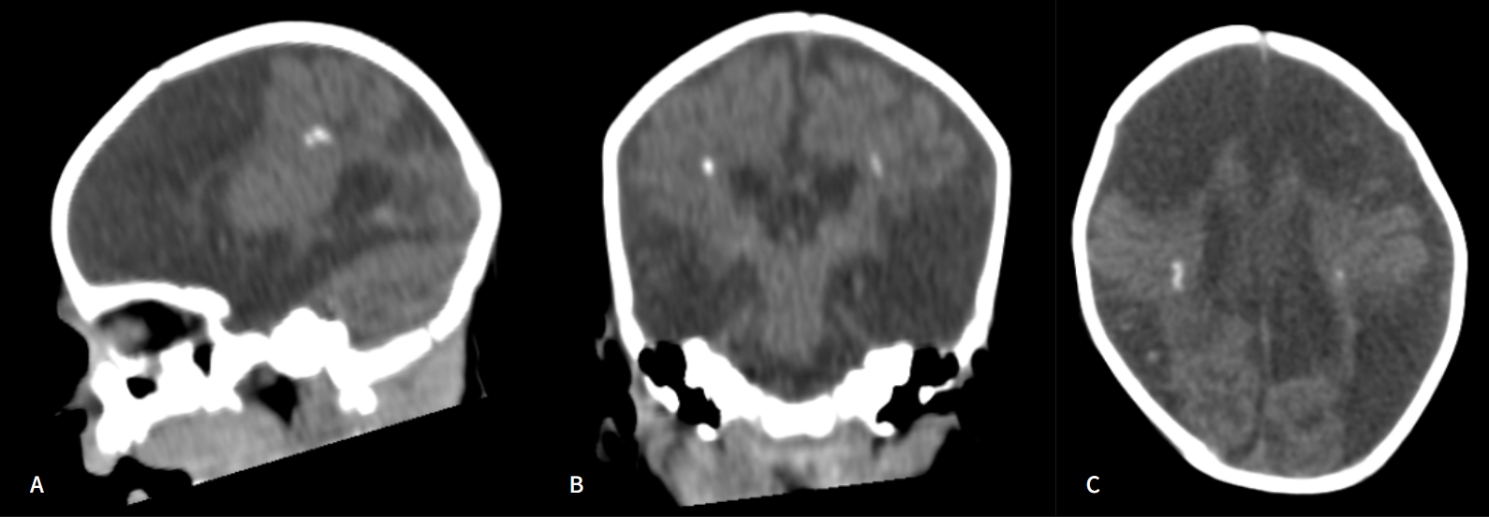
CT scan demonstrating extensive hypodense areas affecting the white matter, multiple areas of parenchymal cystic replacement with sequel-like aspect (cystic leukomalacia), volumetric reduction of the brain and brainstem, and bilateral periventricular foci of calcification; (**A**) Sagittal, (**B**) Coronal, and (**C**) Axial cuts

Afterward, serum immunoglobulin M (IgM) antibodies to HSV-1 and HSV-2 were positive. Immunoglobulin G (IgG) and IgM for toxoplasmosis, HIV, hepatitis B, syphilis, rubella, and cytomegalovirus were all negative. A cranial CT scan demonstrated sequela findings secondary to neonatal hypoxic-ischemic encephalopathy, in addition to areas of calcification in the periventricular white matter and nonhypertensive dilatation of the supratentorial ventricular system (Fig. 4). A new ophthalmological assessment was performed, which showed no progression of necrosis or new lesions in the RE, as well as regression of corneal edema and no lesions in the LE. Intravenous acyclovir was continued for an additional 28 days, followed by oral acyclovir.

A few weeks later, the baby developed abdominal distension, regurgitations, and fecal vomiting. Abdominal ultrasound revealed free fluid in the cavity and intestinal obstruction, requiring surgical intervention. Drainage of voluminous gastric content was performed, and an inflammatory lesion of nonspecific aspect and stenosis in the terminal ileum were identified, requiring resection of 15 centimeters of the ileum. The result of the intraoperative biopsy showed enteric transmural necrosis, indicative of necrotizing enterocolitis.

**Fig. 5:**
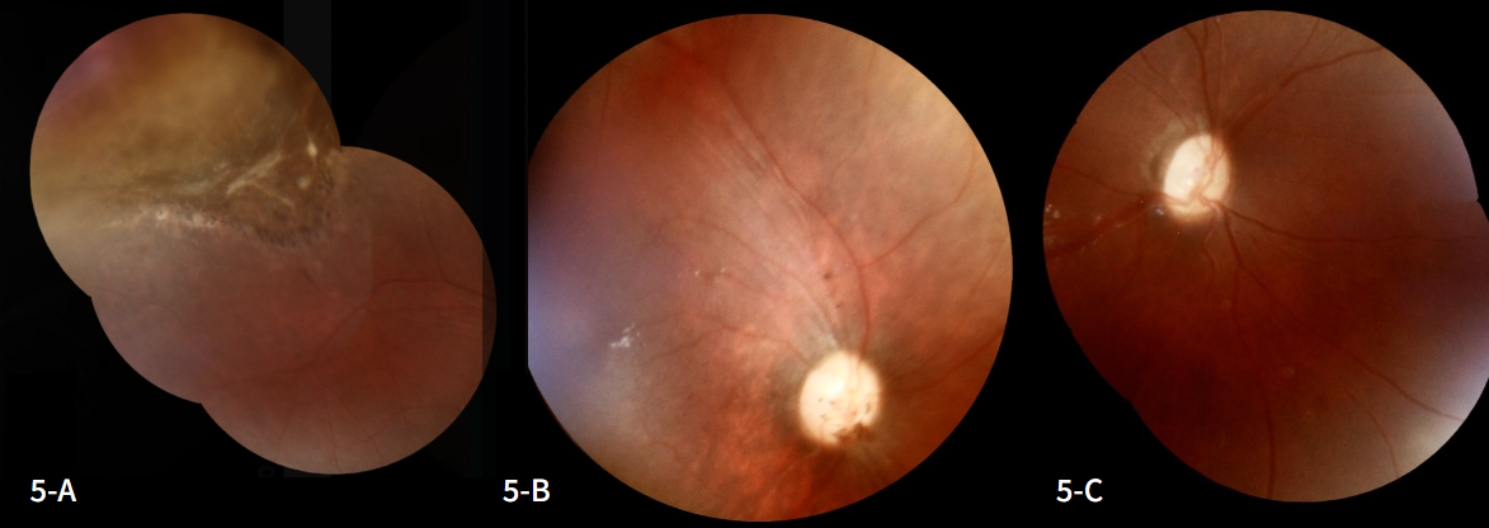
**5-A and 5-B**: Retinal images showing the periphery (**A**) and posterior pole (**B**) of the right eye at the end of treatment; **5-C**: Retinography demonstrating the integrity of the posterior pole of the left eye at the end of treatment

**Fig. 6:**
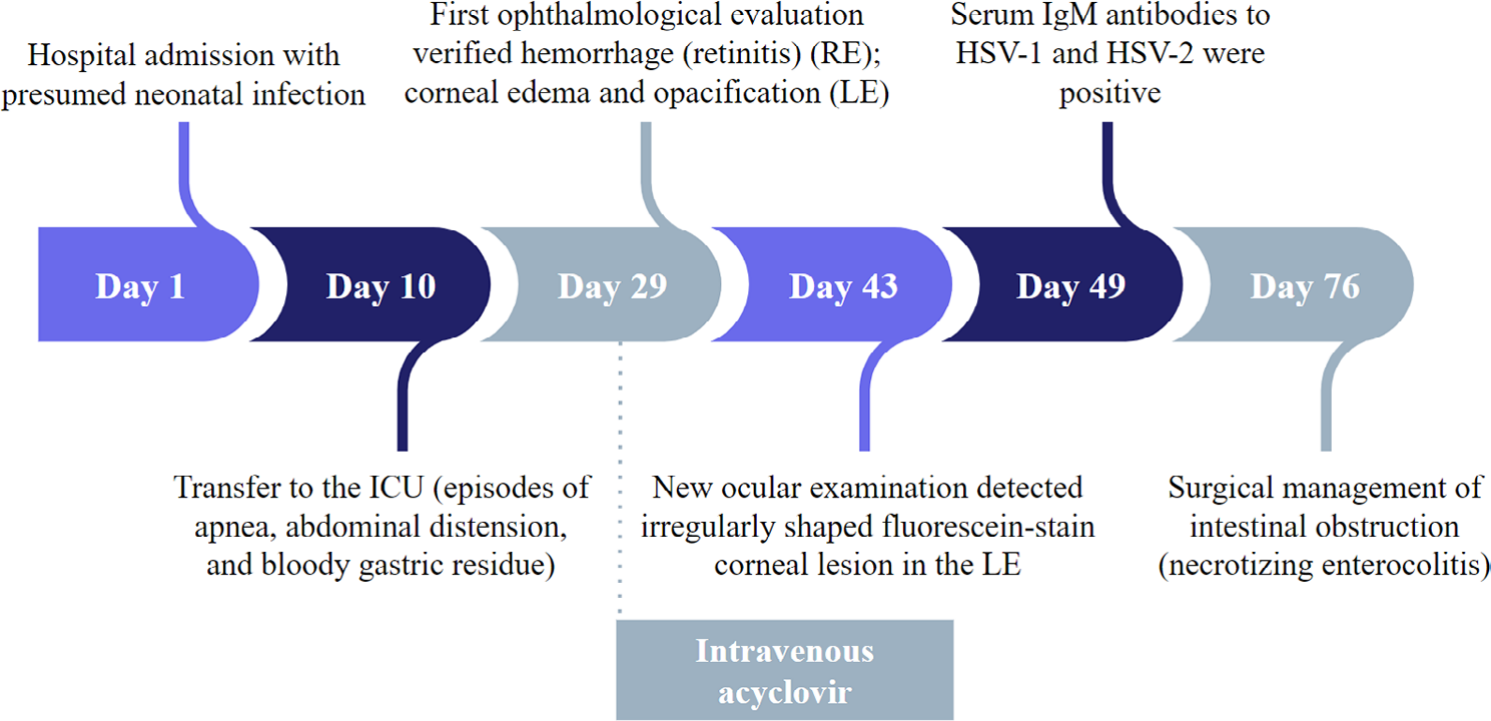
Timeline highlighting the patient’s main clinical features and the role of ophthalmologic evaluation in the diagnosis and proper treatment **Abbreviations**: Intensive care unit (ICU); Right eye (RE); Left eye (LE)

A new ophthalmological examination revealed extensive retinal necrosis in the posterior segment of the RE, with no signs of activity and slight pigmentation near the optic disc in the RE (Fig. 5-A and 5-B). Despite the severity of the newborn’s condition, treatment with acyclovir made it possible to preserve the integrity of the posterior pole of the RE and prevent the spread of infection to the LE (Fig. 5-C). In the patient’s case, adequate antiviral therapy led to an improved prognosis. An overview of the patient’s clinical evolution is depicted in Fig. 6.

At the last ophthalmological follow-up, the patient was 8 months old. In the ocular assessment, the child showed interest in objects > 10 cm in diameter.

## Discussion

Despite being a rare condition, neonatal HSV infection is one of the most important and challenging differential diagnoses in paediatrics [8]. The disease is a costly condition given that possible complications are associated with long hospital stays, continuous monitoring, prolonged drug treatment, and periodic imaging and laboratory tests [10]. Neonatal herpes has high fatality rates, and even with adequate treatment, patients are at risk for persistent skin lesions, long-term neurological disability, and other lasting sequelae [8].

Due to the immaturity of the immune system, prematurely born babies have a higher risk of developing complications from bacterial and viral infections [2]. The risk of transmission of HSV from mother to neonates is greater if the maternal infection is primary since there is insufficient time for transplacental passage of immunoglobulins [11]. In mothers with prolonged infection, it is likely that antibody transfer to the fetus will occur, acting as immunoprophylaxis [12]. Additionally, the risk of neonatal herpes is significantly higher in primary maternal infections near term, as there is no time for an effective immune response against the virus [4].

Congenital herpes simplex is typically diagnosed in newborns after 10 days of age, and the clinical picture comprises the following forms: skin, eye, and mouth (SEM); central nervous system (CNS) impairment; and disseminated disease [3]. Mucocutaneous lesions, usually found as vesicles in the mouth or on the skin, are the most common feature, accounting for approximately 45% of cases [2]. Neonates with neurological involvement account for about 30% of the cases and may manifest irritability, seizures, lethargy, and poor feeding [11].

Disseminated disease is the most severe form, accounting for approximately 25% of infections, and is related to multisystem involvement [3]. In our case, necrotizing enterocolitis stood out as a complication that, although not usually associated with HSV infection, is known to have a multifactorial etiology and may have been precipitated by the condition [13]. HSV manifestations can be mistaken for bacterial sepsis or metabolic disorders due to the patient’s poor appearance and multiorgan symptoms [11].

Differential diagnosis with the group of the most common congenital infections, referred to by the acronym TORCH, remains an aspect of essential consideration, especially in cases with multiple clinical features. However, the classic approach to this investigation, which focuses on serological tests, has been questioned by recent literature, which now advocates a multimodal evaluation that includes radiology, ophthalmology audiology, microbiology, and polymerase chain reaction (PCR) testing, both for infant and placental tissue [14]. Although our case is a satisfactory example of the effectiveness of this approach, we recognize that the applicability of this trend in the literature can be difficult in the majority of low-resource centers, where PCR is not widely available and a multidisciplinary team may not be present [10].

Herpes simplex eye disease can result in cataracts, corneal ulceration, anterior uveitis, vitritis, chorioretinitis, and optic atrophy and is the main cause of corneal visual loss in developed countries [9, 15]. In the acute phase, conjunctivitis is the most frequent ophthalmologic symptom in neonates and is often associated with herpetic epithelial keratitis [16]. Corneal involvement, as in our case, is unilateral in approximately 90% of patients [15]. Considering that there is potential clinical overlap with other etiologies of conjunctival disease, such as gonococcal disease, diagnosis can be challenging [6]. Hence, it is essential to consider HSV infection in cases of neonatal conjunctivitis, especially in patients with an epithelial defect and no improvement after empiric treatment with topical antibiotics [16].

Early diagnosis is essential to improve prognosis but demanding, given the nonspecificity of clinical signs and the challenges of managing neonatal patients. The American Academy of Pediatrics Committee on Infectious Diseases recommends, among other tests, viral culture and PCR of swabs of conjunctiva, CSF, HSV PCR, and serum HSV PCR for evaluation of neonates with suspected diagnosis [11]. Treatment with high-dose intravenous acyclovir (60 mg/kg/day) decreases mortality, reaching 4% in CNS impairment and 30% in disseminated disease, and should extend to at least 21 days in these cases [11]. Afterward, suppressive therapy with oral acyclovir is recommended for a 6-month course [11].

The evolution of the patient demonstrates the importance of considering HSV disease in the range of neonatal infections. The newborn’s clinical picture was marked by multiorgan complications and extensive ocular involvement, with potentially serious long-term sequelae. Additionally, it highlights the role of ophthalmologic assessment in the diagnostic investigation since it guides the clinical judgment toward the correct hypothesis and enables appropriate therapeutic conduct.

## Conclusions

Prevention measures of mother-to-child transmission are essential for decreasing the prevalence and morbimortality rates of congenital herpes. In the management of neonatal HSV, early diagnosis is essential for the timely initiation of antiviral therapy, which decreases the risk of associated complications. Especially in cases of disseminated disease, the neonate should be followed up not only by the pediatrician but also by the ophthalmologist for evaluation of the recurrence of symptoms, sequelae, or side effects of medications. Our report emphasizes that ocular assessment can be central in the diagnostic investigation of this condition and in improving the patient’s prognosis.

## Data Availability

The data supporting the findings of this case report are available from the corresponding author upon reasonable request.

## List of abbreviations

HSV: Herpes simplex virus
LP: Lumbar puncture
CT: Computerized tomography
CSF: Cerebrospinal fluid
RBC: Red blood cells
ICU: Intensive care unit
RE: Right eye
LE: Left eye
HIV: Human immunodeficiency virus
IgM: Immunoglobulin M
IgG: Immunoglobulin G
SEM: Skin, eye, and mouth
CNS: Central nervous system
PCR: Polymerase chain reaction
